# Ultrasound-guided hydrodilatation for adhesive capsulitis: capsule-preserving versus capsule-rupturing technique

**DOI:** 10.1007/s00256-023-04392-7

**Published:** 2023-07-03

**Authors:** Madalena Pimenta, Evangelia E. Vassalou, Michail E. Klontzas, Sofia Dimitri-Pinheiro, Isabel Ramos, Apostolos H. Karantanas

**Affiliations:** 1 Oporto Armed Forced Hospital, University Clinical Center D. Pedro V, Porto, Portugal; 2 Faculty of Medicine, University of Porto, Porto, Portugal; 3 Department of Medical Imaging, University Hospital of Heraklion, Voutes, 71110 Crete, Greece; 4 Department of Radiology, School of Medicine, University of Crete, Voutes Campus, 71110 Heraklion, Greece; 5 Biomedicine Department, Unit of Biochemistry, Faculty of Medicine, University of Porto, Porto, Portugal; 6 Radiology Department, Portuguese Institute of Oncology of Porto – Francisco Gentil EPE, Porto, Portugal

**Keywords:** Frozen shoulder, Adhesive capsulitis, Hydrodilatation, Treatment/ultrasound-guided, Capsular rupture, Predictor

## Abstract

**Objective:**

To compare the clinical efficacy of capsule-rupturing versus capsule-preserving ultrasound-guided hydrodilatation in patients with shoulder adhesive capsulitis (AC). To determine potential factors affecting the outcome over a 6-month follow-up.

**Materials and methods:**

Within a 2-year period, 149 consecutive patients with AC were prospectively enrolled and allocated into (i) group-CR, including 39 patients receiving hydrodilatation of the glenohumeral joint (GHJ) with capsular rupture and (ii) group-CP, including 110 patients treated with GHJ hydrodilatation with capsular preservation. Demographics, affected shoulder, and AC grade were recorded. Disabilities of the Arm, Shoulder and Hand (DASH) questionnaire and visual analog scale (VAS) were used for clinical assessment at baseline/1/3/6 months. Comparisons were performed with Mann-Whitney U test and Kolmogorov-Smirnov test. Linear regression was used to identify predictors of outcome. *P* value < 0.05 defined significance.

**Results:**

DASH and VAS scores in both groups improved significantly compared to baseline (*P* < 0.001) and were significantly lower in the CP compared to CR group at all time-points following intervention (*P* < 0.001). Capsule rupture was a significant predictor of DASH score at all time-points (*P* < 0.001). DASH scores correlated to initial DASH score at all time-points (*P* < 0.001). DASH/VAS scores at 1 month were correlated to the AC grade (*P* = 0.025/0.02).

**Conclusion:**

GHJ hydrodilatation results in pain elimination and functional improvement till the mid-term in patients with AC, with improved outcome when adopting the capsule-preserving compared to the capsule-rupturing technique. Higher initial DASH score is predictive of impaired functionality in the mid-term.

## Introduction

Adhesive capsulitis (AC) is a common disorder presenting with shoulder pain which gradually progresses to global limitation of both active and passive range of motion (ROM). Histopathology involves inflammatory infiltrate of the glenohumeral/subacromial synovium, perivascular lymphocytic reactions, and subsynovial fibrosis, evolving into thickening and contracture of the glenohumeral joint (GHJ) capsule [1].

Despite the fact that AC involving the GHJ is often considered a self-limiting condition, with a reported time-course of up to 2 years, yet not all patients make a full recovery [2]. Adding to this, the rate of complete symptoms’ resolution following conservative treatment has been reported to be as low as 50% [3]. AC associated with systemic secondary causes, mainly including diabetes mellitus, appears to be more resistant to self-recovery and prone to recurrence [4, 5]. This subgroup of patients who experience ongoing pain and disability should be considered as candidates for minimally invasive or operative intervention.

Treatment strategies for AC vary widely; thus, an evidence-based model for the therapeutic management is still lacking [6]. Among other approaches, GHJ hydrodilatation has been shown to be effective in reducing pain and restoring ROM with satisfying short- and long-term outcome in several recent meta-analyses [7–9]. The optimal technique for performing hydrodilatation remains unclear with studies presenting conflicting results. The joint distention volume, the achievement of capsular rupture, the steroid dose, and the anterior or posterior approach through the rotator cuff (RC) interval or posterior GHJ recess, respectively, have been described as the most important among other procedural variables [9–12].

Specifically regarding the necessity of maintaining capsular integrity or not, previous studies comment on the clinical superiority of capsule-rupturing over capsule-preserving technique [13, 14]. However, the injection of diverse volumes without aiming to maximal capsular distension has been regarded as a confounding factor potentially relevant to the less favorable clinical outcome in the latter approach. On the other hand, the capsule-preserving technique, while injecting the maximum volume, has been reported to yield better clinical improvements in pain and joint ROM, compared to capsule rupture [12].

Herein, we sought to compare the short- and long-term clinical efficacy of capsule-rupturing versus capsule-preserving hydrodilatation technique in patients with shoulder AC. In addition, we aimed to determine potential factors affecting the outcome over a 6-month follow-up period.

## Materials and methods

### Participants

The study was conducted according to the Declaration of Helsinki and local regulations. Ethical approval was obtained from the University Hospital Ethics Committee (18,092,021) and written informed consent for participation was obtained from all patients.

**Fig. 1:**
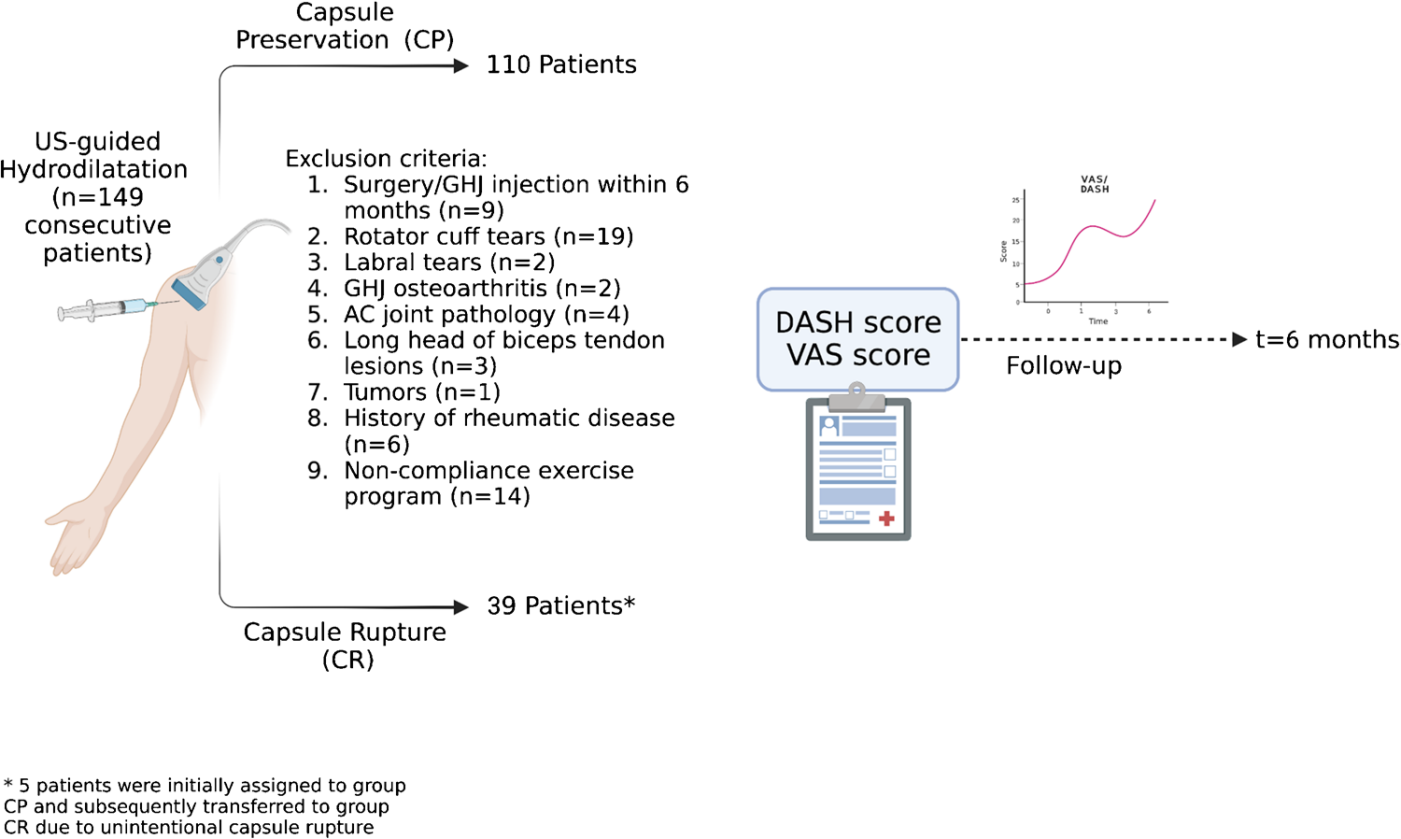
Flow diagram presenting the study design

Within a 2-year period, a total of 209 consecutive patients with AC were prospectively evaluated. AC diagnosis was based on clinical and radiographic criteria and was suggested by (i) symptoms’ duration of > 1 month, (ii) restriction of shoulder ROM shoulder ROM in at least two directions, and (iii) normal GHJ plain radiographs [15]. Restricted ROM was determined by abduction < 80°, forward flexion < 130°, and external rotation < 30° [16]. According to physical examination findings and reported symptoms, the disease was classified into three grades (I, freezing; II, frozen; III, thawing) at presentation [17]. Exclusion criteria included the following: previous shoulder surgery/previous GHJ injection within 6 months (*n* = 9), rotator cuff tears (*n* = 19), labral tears (*n* = 2), GHJ degenerative osteoarthritis (*n* = 2), acromioclavicular joint pathology (*n* = 4), long head of biceps tendon tears (*n* = 3), shoulder bone and soft tissue tumors (*n* = 1), history of rheumatic disease (*n* = 6), and those lost during follow-up or non-complying with the home-based exercise program (*n* = 14). Non-eligible patients were isolated based on the combination of clinical assessment, diagnostic US examination, and MR imaging findings in selected cases (*n* = 21). MR imaging was particularly evaluated for the confirmation of labral tears, GHJ degenerative osteoarthritis, presence of shoulder bone and soft tissue tumors and the assessment of the intra-arcticular part of the long head of biceps tendon. The study group comprised 149 patients (Fig. 1).

Patients were allocated into 2 groups according to the performed US-guided hydrodilatation technique based on the preference of the referring physician and/or patient: (i) group-CR, including those receiving hydrodilatation of the GHJ with termination of the procedure following capsular rupture and (ii) group-CP, including patients treated with hydrodilatation of the GHJ with termination of the procedure prior to capsular rupture. Demographics, the affected shoulder (dominant/non-dominant), and the grade of AC were recorded for all patients at presentation. Potential adverse reactions related to treatment were also recorded.

### Clinical evaluation and follow-up

All patients were clinically assessed by four senior orthopedic surgeons at baseline, 1 month, 3 months, and 6 months post-hydrodilatation. The Disabilities of the Arm, Shoulder and Hand (DASH) questionnaire, ranging from 0 (no disability) to 100 (most severe disability), was used for the evaluation of functional impairment and associated pain. DASH scores were calculated using the online tool found at https://orthotoolkit.com/dash/. A visual analog scale (VAS) ranging from 0 (no pain) to 10 (worst pain ever felt) was used to assess the intensity of shoulder pain at the same time-points. A VAS score of 0 or 1 at the final time point was used to define complete pain resolution, as previously described [18].

### US-guided intervention

All procedures were performed by two senior radiologists with 37-year and 12-year experience on musculoskeletal imaging and intervention. Both operators were trained to perform the procedure in an identical manner. A high-frequency linear array probe (6–15 MHz) in a Siemens ACUSON Sequoia or a GE Logiq E9 system was used.

A diagnostic US survey preceded the interventional procedure in all patients. The examination protocol was adhered to the guidelines proposed by the European Society of Musculoskeletal Radiology [19]. In this context, the rotator cuff, posterior shoulder structures/posterior GHJ recess, and the acromioclavicular joint were routinely evaluated.

**Fig. 2:**
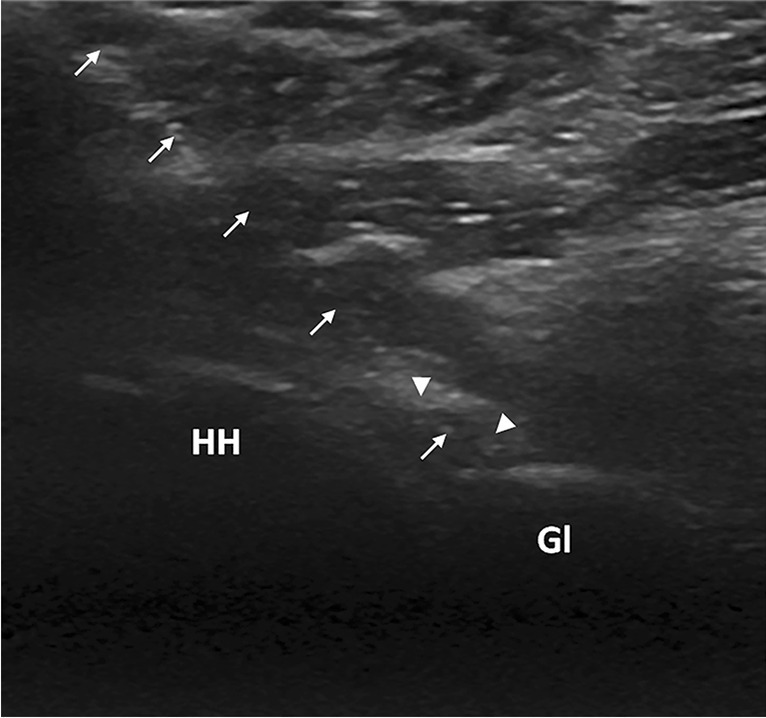
Glenohumeral joint hydrodistension in a 52-year-old female via the posterior approach. Sonographic image showing the needle’s lateral-to-medial course (arrows) ending within a slightly distended posterior joint recess (arrowheads). HH, humeral head; Gl, glenoid

US-guided hydrodilatation was performed with the patient in the semi-prone position, the affected shoulder lying uppermost and the ipsilateral shoulder and elbow joints at 90° of extension and flexion, respectively. A supporting pillow was placed under the affected shoulder, in order to maximize comfort and ensure stability. All the procedures were performed under full sterile and aseptic conditions, using sterile gloves, probe covers and standard skin preparation, following the application of sterile gel. After that, the US transducer was positioned along the long axis of the myotendinous junction of the infraspinatus tendon (in-plane approach) just inferior to the scapular line. The contours of the posterior glenoid rim, the posterior glenoid labrum and the posterior part of the humeral head, which had to been shown on a single US image, served as important landmarks. Then, a 18-gauge spinal needle, allowing higher pressure during injection, was advanced under constant US guidance in a lateral-to-medial direction, until the needle tip entered the GHJ (Fig. 2) [20].

Once the proper injection spot had been determined, a solution composed of 3 mL of lidocaine 1%, 3 mL of ropivacaine 0.25%, and 1 mL of betamethasone 40 mg/mL, followed by infusion of up to 40 mL of normal saline were injected to distend or rupture the joint capsule, as required.

**Fig. 3:**
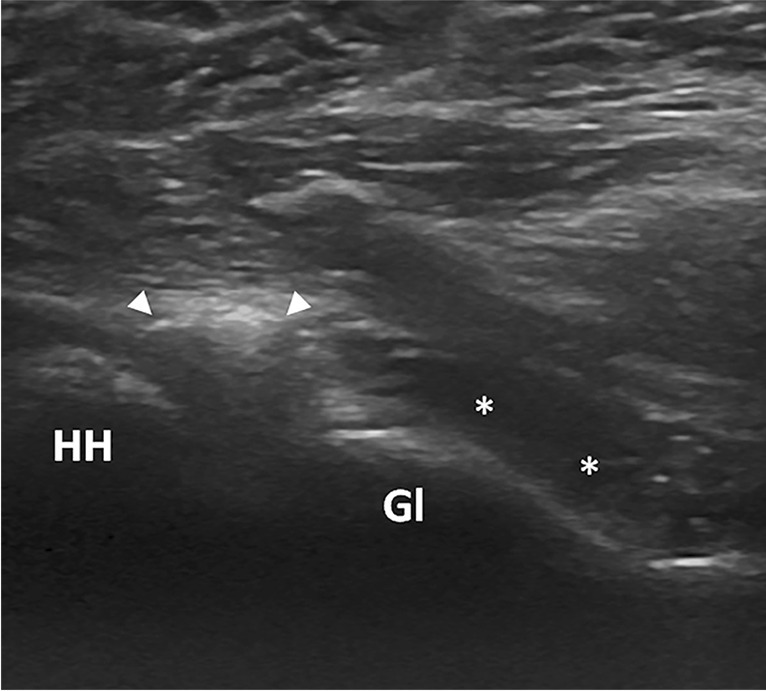
Glenohumeral joint hydrodistension with capsular rupture in a 58-year-old female. Sonographic image shows a distended posterior joint recess (arrowheads), with extraarticular fluid (*) extending medially following capsular rupture. HH, humeral head; Gl, glenoid

For patients in group-CR, the procedure was terminated when there was either (i) sudden loss of resistance in injecting the fluid or (ii) a feeling of discomfort and/or pain in the axilla or the medial aspect of the humerus. Capsular rupture was further confirmed by the presence of extracapsular leakage of the injected fluid into the periarticular soft tissues (Fig. 3). For patients in group-CP, the procedure was terminated either when (i) a maximum resistance did not allow any further volume to be injected or (ii) there was unintentional capsular rupture. The patients in group-CP who had sustained unintended capsular rupture were assigned to group-CR.

For eliminating confounding effects, participants were not permitted to take pain-relieving medication beyond the first 5 days after the intervention or undertake other manual treatments or interventional procedures, along the duration of the study.

### Standard post-procedural care

All participants were instructed how to perform a home-exercise program, including Codman exercises, table-lean passive stretches and wall-climbing exercise with the fingers. The exercise program was initiated the day after the interventional procedure with a subsequent routine 3-month course. Patients were asked to regularly perform the program, at least 5 times per day, starting with five and gradually increasing up to 10 repetitions of each specific exercise.

### Statistical analysis

Statistical analysis was performed with the use of SPSS Statistics v 29 (IBM Corp., Armonk, NY). Descriptive statistics were used to analyze demographical data at baseline for our patient cohort and values were expressed as frequencies and mean ± SD. Mean comparisons between groups at each time points were performed with the use of the non-parametric Mann-Whitney *U* test. Predictors of DASH and VAS scores at 1, 3, and 6 months were examined with univariate linear regression models, and those achieving significance in univariate analysis were used to construct a multivariate linear regression model to identify independent predictors of VAS and DASH scores [18]. Sample size estimation given a 1:3 group-CR/group-CP ratio, power at 0.8 and significance at 0.05, yielded a minimum sample size of 60 patients (15 for group-CR and 45 for group-CP). *P* value lower than α = 0.05 was used to define significance.

## Results

### Patient baseline characteristics

Our cohort consisted of 149 patients (43 male, 106 female) with a mean age of 53.3 ± 10.1 years. Five patients who were initially assigned to group-CP, sustained unintended capsular rupture and were included in group-CR. Group-CR included 39 patients (13 male, 26 female, mean age 57 ± 12 years) and group-CP a total of 110 patients (30 male, 80 female, mean age 52 ± 9 years). No significant difference was found between the mean age, sex and grade of AC at presentation, between group-CR and group-CP. The dominant and non-dominant upper limb was affected in 90 and 59 patients, respectively. At presentation, 54, 66, and 29 patients had stage I, II, and III AC, respectively. All patients, in both groups, reported full compliance with the home exercise program. Patient demographics and baseline characteristics are presented in Table 1.

**Table 1:** Patients’ demographics and baseline characteristics for both study groups

	Group-CP	Group-CR
Number of enrolled patients	110	39
Gender	30 males, 80 females	13 males, 26 females
Affected side (dominant/non-dominant)	69 dominant/41 non-dominant	21 dominant/18 non-dominant
Mean age ± SD	52 ± 9 years (range, 25–75 years)	57 ± 12 years (range, 37–81 years)
Number of patients with each stage of adhesive capsulitis (I/II/III)	6 stage I/29 stage II/4 stage III	48 stage I/37 stage II/25 stage III
Mean injection volume ± SD	24.8 ± 5 mL	36.2 ± 8 mL

I/II/III refers to the “freezing,” “frozen,” and “thawing” stage of adhesive capsulitis, respectively

*CP* capsular preservation, *CR* capsular rupture, *SD* standard deviation

### Treatment outcome

**Fig. 4:**
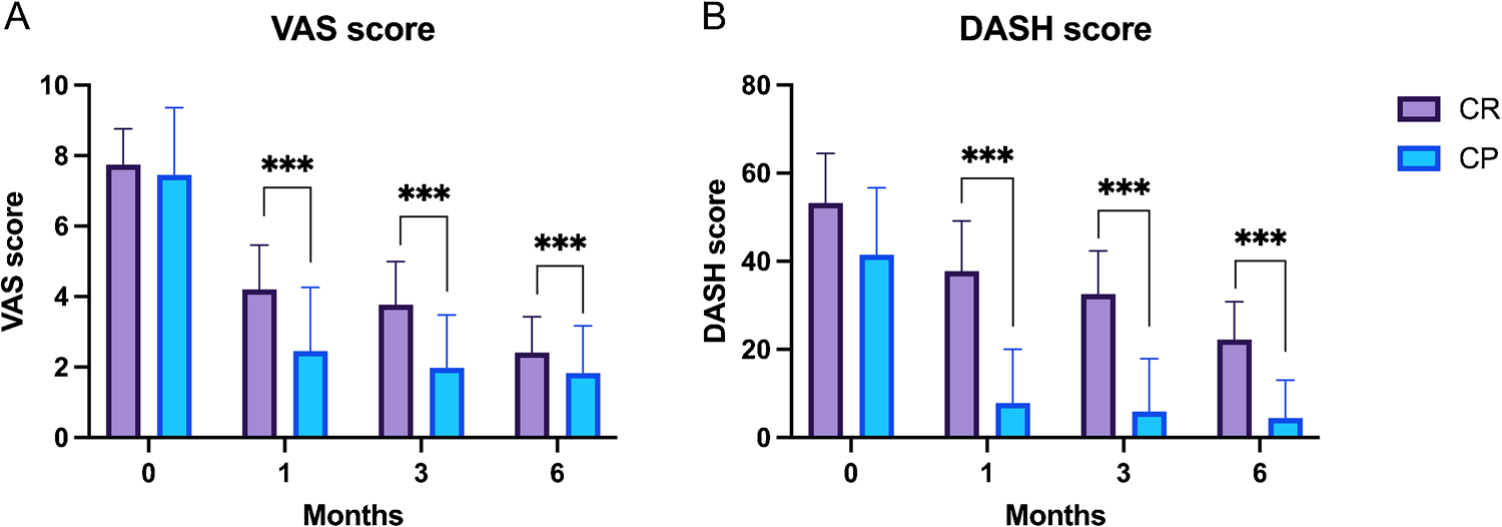
Visual analog scale (VAS) (**A**) and Disabilities of the Arm Shoulder and Hand (DASH) scores (**B**) over time in the capsule rupture (CR) and capsule preservation (CP) groups; ***P < 0.05

No significant complications related to US-guided hydrodilatation were recorded over the study course. DASH and VAS scores in both groups improved significantly at all follow-up time-points compared to baseline (*P* < 0.001 for all comparisons). DASH and VAS scores were lower in the CP group compared to the CR group (*P* < 0.001 for all comparisons) at all time-points following the intervention (Fig. 4).

Predictors of DASH and VAS scores at all follow-up time-points were examined by linear regression at a univariate and multivariate level. Univariate analysis indicated that capsule rupture was a significant predictor of DASH and VAS scores at all time-points (*P* < 0.001). At month 1, DASH and VAS scores were positively corelated to the grade of AC (*P* = 0.025, *P* < 0.001, respectively). DASH scores at baseline were significantly correlated with DASH scores at all follow-up time-points (*P* < 0.001 for all comparisons). Multivariate analysis indicated that capsule rupture is an independent predictor of DASH score at all time-points (*P* < 0.001) and VAS scores at month 1 and month 3 (*P* < 0.05). The results of univariate and multivariate linear regression analysis of DASH and VAS are provided in Tables 2, 3, and 4.

**Table 2:** Univariate linear regression analysis of DASH scores after US-guided hydrodistention

	Time point	Variable	*R*^2^	B coefficient	95% CI of B	*P* value
Follow-up time points	1 month	Capsule Rupture	0.55	29.992	25.567 to 34.417	< 0.001 #
		Initial DASH score	0.199	0.532	0.354 to 0.710	< 0.001 #
		Initial VAS score	0.029	1.954	0.296 to 3.612	0.021 #
		Sex	0.005	2.662	− 3.720 to 9.044	0.411
		Age	0.013	0.201	− 0.085 to 0.487	0.166
		AC grade	0.034	4.49	0.572 to 8.408	0.025 #
	3 months	Capsule Rupture	0.52	26.787	22.588 to 30.985	< 0.001 #
		Initial DASH score	0.155	0.433	0.264 to 0.601	< 0.001 #
		Initial VAS score	0.003	0.489	− 1.06 to 2.038	0.533
		DASH score at 1 month	0.536	0.672	0.57 to 0.775	< 0.001 #
		VAS score at 1 month	0.19	3.868	2.565 to 5.172	< 0.001 #
		Sex	0	− 0.55	− 6.426 to 5.326	0.854
		Age	0.01	0.165	− 0.098 to 0.428	0.216
		AC grade	0.013	2.608	− 1.029 to 6.244	0.159
	6 months	Capsule Rupture	0.462	17.87	14.73 to 21.01	< 0.001 #
		Initial DASH score	0.071	0.206	0.082 to 0.331	0.001 #
		Initial VAS score	0.015	0.821	− 0.268 to 1.909	0.138
		DASH score at 1 month	0.331	0.374	0.287 to 0.46	0.001 #
		VAS score at 1 month	0.126	2.231	1.274 to 3.188	< 0.001 #
		DASH score at 3 months	0.351	0.419	0.326 to 0.512	< 0.001 #
		VAS score at 3 months	0.266	3.671	2.677 to 4.664	0.001 #
		Sex	0.009	− 2.411	− 6.548 to 1.725	0.251
		Age	0.019	0.156	− 0.029 to 0.341	0.097
		AC grade	0.002	0.739	− 1.847 to 3.325	0.573

#: statistically significant value

**Table 3:** Univariate linear regression analysis of VAS scores after US-guided hydrodistention

	Time point	Variable	*R*^2^	B coefficient	95% CI of B	*P* value
Follow-up time points	1 month	Capsule Rupture	0.175	1.751	1.131 to 2.37	< 0.001 #
		Initial DASH score	0.131	0.044	0.025 to 0.064	< 0.001 #
		Initial VAS score	0.083	0.309	0.142 to 0.477	< 0.001 #
		Sex	0.019	0.564	− 0.091 to 1.219	0.091
		Age	0.001	0.006	− 0.023 to 0.036	0.673
		AC grade	0.036	0.480	0.075 το 0.885	0.02 #
	3 months	Capsule Rupture	0.234	1.787	1.26 to 2.314	< 0.001 #
		Initial DASH score	0.077	0.03	0.013 to 0.048	< 0.001 #
		Initial VAS score	0.009	0.088	− 0.066 to 0.241	0.261
		DASH score at 1 month	0.331	0.053	0.04 to 0.065	< 0.001 #
		VAS score at 1 month	0.291	0.476	0.355 to 0.597	< 0.001 #
		Sex	0	0.076	− 0.508 to 0.66	0.796
		Age	0.001	− 0.005	− 0.032 to 0.021	0.689
		AC grade	0.006	0.168	− 0.195 to 0.531	0.362
	6 months	Capsule Rupture	0.04	0.583	0.115 to 1.051	0.015 #
		Initial DASH score	0	0	− 0.014 to 0.015	0.95
		Initial VAS score	0.007	0.063	− 0.059 to 0.185	0.31
		DASH score at 1 month	0.053	0.017	0.005 to 0.028	0.005 #
		VAS score at 1 month	0.078	0.196	0.086 to 0.306	< 0.001 #
		DASH score at 3 months	0.044	0.017	0.004 to 0.029	0.01 #
		VAS score at 3 months	0.137	0.294	0.174 to 0.414	< 0.001 #
		Sex	0.002	− 0.126	− 0.589 to 0.336	0.59
		Age	0.014	0.015	− 0.005 to 0.036	0.147
		AC grade	0.002	− 0.07	− 0.358 to 0.218	0.633

#: statistically significant value

**Table 4:** Multivariate linear regression analysis of DASH and VAS score predictors after US-guided hydrodistention

	Time point	Variable		*R*^2^	B coefficient	95% CI of B	*P* value
DASH score	1 month			0.593			
		Initial DASH score			0.211	0.053 to 0.369	0.009
		Initial VAS score			0.4	− 0.871 to 1.670	0.535
		Capsule Rupture			26.982	22.317 to 31.647	< 0.001 #
		AC grade			1.606	− 1.215 to 4.427	0.065
	3 months	Initial DASH score		0.626	0.087	− 0.04 to 0.215	0.177
		VAS score at 1 month			-1.209	− 2.645 to 0.277	0.098
		DASH score at 1 month			0.493	0.29 to 0.695	< 0.001 #
		Capsule Rupture			13.372	7.423 to 19.321	< 0.001 #
	6 months	Initial DASH score		0.51	0.009	− 0.096 to 0.114	0.868
		DASH score at 1 month			0.103	− 0.082 to 0.289	0.271
		VAS score at 1 month			− 0.075	− 2.106 to 0.596	0.271
		DASH score at 3 months			− 0.79	− 0.291 to 0.134	0.465
		VAS score at 3 months			2.278	0.593 to 3.964	0.008 #
		Capsule Rupture			13.823	8.608 to 19.038	< 0.001 #
VAS score	1 month			0.266			
		Initial DASH score			0.015	− 0.006 to 0.037	0.166
		Initial VAS score			0.201	0.025 to 0.377	0.025 #
		Capsule Rupture			1.488	0.841 to 2.135	< 0.001 #
		AC grade			0.209	− 0.183 to 0.6	0.293
	3 months	Initial DASH score		0.391	0	− 0.016 to 0.016	0.957
		DASH score at 1 month			0.016	− 0.009 to 0.042	0.209
		VAS score at 1 month			0.278	0.097 to 0.459	0.003 #
		Capsule Rupture			0.84	0.091 to 1.59	0.028 #
	6 months	DASH score at 1 month		0.18	0.004	− 0.022 to 0.03	0.746
		DASH score at 3 months			0.035	− 0.065 to − 0.006	0.018 #
		VAS score at 1 month			0.029	− 0.158 to 0.216	0.763
		VAS score at 3 months			0.465	0.233 to 0.697	< 0.001 #
		Capsule Rupture			0.519	− 0.215 to 1.252	0.165

#: statistically significant value

## Discussion

Herein, we compared the clinical outcome of capsule-rupturing versus capsule-preserving hydrodilatation technique in patients with shoulder AC at various follow-up time-points, till 6 months. To the best of our knowledge, this is the first study assessing the efficacy and comparing these procedural variables in the mid-term. Both approaches showed significant improvement in terms of pain and disability; however, capsule preservation showed clinical superiority in terms of pain improvement and disability both in the short- and mid-term, compared to the capsule rupturing approach. Additionally, capsule rupture appeared to be an independent predictor of impaired functionality at all time-points and existence of pain at 1 and 3 months. Furthermore, advanced AC grade correlated to impaired functionality and pain in the short-term (1 month post-treatment). Finally, DASH score at presentation was a predictor of impaired functional status at all time-points.

Although various treatment options have been described for AC, their clinical efficacy remains to be further evaluated in order to establish an evidence-based therapeutic pathway [8, 21]. Conservative treatment consisting of certain physical therapy techniques, nonsteroidal anti-inflammatory drugs and intraarticular steroid injections is strongly recommended as a first-line approach especially during the painful stage of AC [22, 23]. In the setting of failed conservative treatment, GHJ hydrodilatation represents another minimally invasive therapeutic option. The method, especially when combined with intra-articular steroid administration, has been reported to be effective in reducing pain and restoring ROM in AC with satisfying short- and long-term outcome [24]. In our study, all patients, irrespective of sustaining capsular rupture, showed significant improvement in shoulder pain and functional status, both in the short and long term.

A previous study by Kim et al. has shown enhanced effect of hydrodilatation by preserving the capsule in patients with AC in the short term [12]. Improvement of pain and ROM was significantly greater in the capsule-preserved group at both follow-up time-points. Similarly, our results confirmed the adverse effect of hydrodilatation-induced capsular rupture on the improvement of pain and shoulder disability in the short- (1 month) and mid-term (3 and 6 months). However, the correlations between capsular rupture and clinical scores in that study were much weaker compared to our correlations at all relevant time-points. This may be due to existing methodological differences between the two studies, assessing different disability scores (LOM score/Kim et al. versus DASH score/present study) and including different numbers of participants (54 patients/Kim et al. versus 149 patients/present study). Additionally, the multivariate analysis performed herein suggested capsular rupture as an independent predictor of impaired functionality at all time-points and existence of pain, which further strengthens the validity of our outcomes. Our results are also in accordance with the outcomes of Yang et al. indicating that capsule rupture during hydrodilatation was not a significant predictor of clinical improvement at 2 months post-treatment [25].

Impaired functionality at presentation indicated by higher initial DASH scores was a predictor of worse functional status at all time-points. This is in accordance with the results of Bell et al., suggesting that more severely affected shoulders at baseline, although showing greater absolute measured increase in ROM following hydrodilatation, still suffered a higher degree of impaired functionality at 2 months post-treatment, compared to less severely affected patients [26]. However, given the technical differences between the two studies, with capsular rupture representing the end-point of hydrodilatation in the study of Bell et al., such a comparison should be interpreted with caution. In general, the predictors of clinical outcome after GHJ hydrodilatation in patients with AC have not been substantially investigated. Determination of such predictors is of clinical importance as it may aid in patient individualized management by isolating those with AC who are most likely to benefit from hydrodilatation. The mean capsule-preserving intraarticular fluid volume during hydrodilatation in AC patients has been reported to be 25.1 ± 6.9 mL [27]. Thus, for an effective procedure, while maintaining the capsular integrity, the optimal minimal injected volume is regarded to be approximately 18 mL [27]. Additionally, Ogul et al. found that the mean GHJ capacity in patient with AC was 22.52 cm^3^ versus 26.01 cm^3^ in the control group [28]. Considering the above, a total instilled volume of up to 47 mL in the capsule-preserved subgroup of our study is regarded sufficient for ensuring continuous and maximal expansion of the contracted capsule causing elimination of adhesions which has been linked to improved ROM [29] On the other hand, capsular rupture leads to extraarticular leakage of the injected steroid which may account for the less favorable outcome in these patients.

In this study, real-time intraarticular pressure monitoring was not performed. Intraarticular placement of disposable sensors for constant pressure observation during hydrodilatation in AC has been adopted in previous studies, yielding a 3-phased pressure-volume curve [12, 30]. Beyond research purposes, the main clinical usefulness of pressure monitoring is to predict the time of capsule rupture and alert for termination of the procedure immediately before this time-point, while achieving the maximal pre-rupture intraarticular pressure. However, despite the fact that specific characteristics of this curve together with cutoff values of intraarticular pressure measurements have been suggested to serve as “pre-rupture” signs, these criteria may fail to preclude capsular rupture in about 15% of patients [12].

Our study has specific strengths and limitations. Its prospective nature and the long-term follow-up could be regarded as important strengths. However, specific limitations should be mentioned. Firstly, no-treatment control group was defined which would allow a comparison of our study population with the natural history of AC. Secondly, the study was not tailored to patients with a specific stage of AC. In this regard, sporadic studies have suggested that the outcome of hydrodilatation is stage-dependent and such an approach is more effective and should be reserved for patients with stage II AC [29]. However, this hypothesis has not been further confirmed and represents a field of potential further research. Thirdly, the fact that patients’ allocation into each group, was based on the preference of the referring physician and/or patient to undergo US-guided hydrodilatation with or without capsular rupture, has resulted to imbalance between the participants of the two groups. However, this has been accounted in the sample size estimation to ensure robust statistics. Finally, potential compliance issues with the home-based, non-supervised exercise program may have an impact on our results, although all patients reported full compliance.

In conclusion, hydrodilatation of the GHJ results in pain elimination and functional improvement in patients with AC, with achievement of improved outcome when adopting the capsule-preserving compared to the capsule-rupturing technique. Hydrodilatation-induced capsular rupture appears to have an adverse effect on shoulder functionality and pain and represents an independent predictor of worse functional status both in the short- or mid-term. DASH score at presentation was a predictor of impaired functional status at all time-points.
